# A novel methodology for strengthening human rights based monitoring in public health: Family planning indicators as an illustrative example

**DOI:** 10.1371/journal.pone.0186330

**Published:** 2017-12-08

**Authors:** Sofia Gruskin, Laura Ferguson, Shubha Kumar, Alexandra Nicholson, Moazzam Ali, Rajat Khosla

**Affiliations:** 1 Program on Global Health and Human Rights, Institute for Global Health, University of Southern California, Los Angeles, California, United States of America; 2 Department of Reproductive Health and Research, World Health Organization, Geneva, Switzerland; Western Sydney University, AUSTRALIA

## Abstract

**Objective:**

The last few years have seen a rise in the number of global and national initiatives that seek to incorporate human rights into public health practice. Nonetheless, a lack of clarity persists regarding the most appropriate indicators to monitor rights concerns in these efforts. The objective of this work was to develop a systematic methodology for use in determining the extent to which indicators commonly used in public health capture human rights concerns, using contraceptive services and programmes as a case study.

**Methods:**

The approach used to identify, evaluate, select and review indicators for their human rights sensitivity built on processes undertaken in previous work led by the World Health Organization (WHO). With advice from an expert advisory group, an analytic framework was developed to identify and evaluate quantitative, qualitative, and policy indicators in relation to contraception for their sensitivity to human rights. To test the framework’s validity, indicators were reviewed to determine their feasibility to provide human rights analysis with attention to specific rights principles and standards.

**Findings:**

This exercise resulted in the identification of indicators that could be used to monitor human rights concerns as well as key gaps where additional indicators are required. While indicators generally used to monitor contraception programmes have some degree of sensitivity to human rights, breadth and depth are lacking.

**Conclusion:**

The proposed methodology can be useful to practitioners, researchers, and policy makers working in any area of health who are interested in monitoring and evaluating attention to human rights in commonly used health indicators.

## Introduction

Over the last few years the field of public health has seen a rise not only in general rhetorical attention to human rights, but in the number of initiatives that work to incorporate human rights into public health practice, policies and programmes [1]. In 2015, the United Nations Sustainable Development Goals (SDGs) outlined multiple goals, which touch on economic and social rights, and affirm principles of inclusivity, non-discrimination and accountability [2,3]. The promotion and protection of human rights has been shown to be key to effective delivery of quality health services and ensuring accountability in public health programmes, including assurance and evidence of how governments and the international community are fulfilling their relevant human rights obligations. Incorporating due attention to human rights in all aspects of programming, including monitoring and evaluation, is key to service delivery, as well as progress towards the fulfillment of health-related rights obligations [1,4, 5]. Overcoming legal, policy and system barriers requires identification, careful analysis and subsequent modification–through laws, policies and practices that are consonant with human rights–with the ultimate aim of improving access and use of needed services through sound public health practice and the promotion and protection of human rights. Identifying and implementing indicators sensitive to human rights principles and standards is a necessary step to achieve this. While many agencies and organizations work to integrate human rights into public health policies and programs, as rigorous tools or resources in this area remain limited, they often struggle to monitor and evaluate how well human rights are actually being addressed or fulfilled in these efforts. Despite positive steps, a lack of clarity persists regarding the most appropriate indicators [6] for monitoring the promotion or violation of rights in public health efforts [4], contributing to general uncertainty about the added value of incorporating human rights into public health practice.

Evaluation methods and indicators that specifically capture human rights concerns are not well developed, and those that exist are often used inconsistently [7,8,9]. For an indicator to be valid from both a health and human rights perspective, irrespective of why it was initially constructed, it is essential to determine the extent of its human rights sensitivity and its validity in public health terms [4]. The respect, protection and fulfillment of human rights is necessary to achieve desired public health outcomes. To support organizations interested in using human rights to strengthen public health programming, the World Health Organization (WHO)/Human Reproduction Program (HRP) in partnership with the Program on Global Health and Human Rights at the University of Southern California set out to develop and test a methodology to analyze the human rights sensitivity of indicators in contraceptive programming standardly used in public health practice. In an environment of indicator proliferation, this methodology explicitly sought to identify indicators that were already being used in practice and could most adequately meet human rights-related information needs to aid in decision-making with respect to human rights in contraceptive programming.

This article presents the methodology and lessons learned from this work, using indicators related to contraception as a case example. Importantly, this process, which can be replicated for any health topic, not only produces a list of prioritized indicators sensitive to human rights principles and standards, but also provides insights into key human rights concerns insufficiently addressed in programmes or the indicators used to assess them. The broader implications of this approach to further the analysis and integration of human rights across public health monitoring, evaluation and implementation efforts are explored in the discussion section.

## Methodology

This research sought to develop a methodology for analyzing the human rights sensitivity of public health indicators. To determine the validity of the proposed methodology, quantitative, qualitative and policy indicators commonly used at global level and by national health information systems for the monitoring and evaluation of contraception programming at country, regional and global levels were reviewed. A series of stages and steps as described below were undertaken to both develop and test the methodology.

An expert advisory group (EAG) composed of key representatives from the WHO and other international organizations, ministries of health, non-governmental organizations, and academic institutions, alongside a multidisciplinary research team of health researchers and human rights lawyers with expertise in human rights, sexual and reproductive health, and monitoring and evaluation were recruited as an initial step towards identifying a prioritized list of indicators. Close collaboration ensued between WHO, the research team and the EAG for the life of the project. Additionally, a meeting of regionally and disciplinarily diverse stakeholders representing organizations including governmental agencies, non-governmental organizations, multilateral agencies, advocacy groups, academic and research institutions was organized partway through the study to review the methodology and preliminary findings resulting in refinement of the methodology and indicators described below.

### Identification and review of standards, sources, and key gaps to inform analysis

The approach used to identify, evaluate, select and review indicators for their human rights sensitivity built on previous work which identified relevant human rights principles based on international standards (S1 Appendix). These human rights principles and standards find resonance in recommendations made to States by human rights treaty monitoring bodies and in international consensus documents. While directly relevant to the provision of contraceptive programming, they are applicable to the delivery of programming with respect to other health topics as well.

Using these human rights principles and standards, a 2014 WHO report identified 12 existing quantitative indicators used in contraceptive programming, which taken together could begin to shed light on human rights concerns [10]. Through consultation with the EAG, the shortcoming of relying purely on quantitative indicators was noted and it was agreed that for the present exercise a relevant mix of quantitative, qualitative and policy indicators would be used to maximize comprehensiveness and ensure that attention to rights in relevant indicators was captured to the extent possible. Therefore, indicators currently in use that employ qualitative methodologies and policy indicators to show gaps between laws and policies and the ways in which relevant services are delivered were identified, alongside additional quantitative indicators determined by the EAG to be of relevance to this exercise. Bringing these sources together resulted in an initial list of 208 indicators to review.

Through consultation with the EAG, it was agreed that the criteria described in S1 Table, would form the basis of the analytic framework used in a step-wise manner to analyze the 208 indicators for their sensitivity to human rights.

S1 Table describes the information included and the domains for analysis as they were applied to each indicator. The first column of S1 Table contains the exact language of the indicator chosen for review and any relevant explanatory text from the source. The second column contains an assessment by researchers of the human rights principles and standards potentially addressed by the indicator. Using legally grounded definitions of each human rights standard and principles (see S1 Appendix), the rationale underlying the selection of each right potentially addressed by the indicator is noted. The third column contains an assessment of whether the link(s) between the indicator and the human rights standard(s) are explicit and/or implicit. An indicator was considered to have an explicit linkage if it specifically included a human rights principle or standard in its definition. Conversely, an indicator was considered to have an implicit linkage if it was thought to potentially measure an aspect of human rights, even if rights are not explicitly addressed in its definition. The fourth column provides substantive detail to explain the determination of whether the linkage is considered to be explicit or implicit. The fifth column contains an assessment of whether the indicator captures user perspectives (reflecting another important human rights dimension). The sixth column contains an assessment of whether the indicator includes a focus on inequalities and/or a specific population that faces challenges in accessing and using the relevant health service (an indicator was considered to focus on inequalities and/or a specific population if a particular population or group was explicitly addressed in its definition). Finally, the last column contains an assessment of whether the indicator lends itself to disaggregation and therefore investigation for potential accordance with national non-discrimination law. This analytic framework assigns a column for each of the domains and a row for each indicator analyzed. This tabular format allowed for easy analysis and comparison between indicators and across columns and rows.

Researchers performed independent analyses of each indicator’s sensitivity to human rights concerns and results were triangulated across the team and refined through focused discussion.

In the example used, these steps produced a completed table containing the analysis of all 208 indicators, providing a broad overview of the potential for human rights sensitivity of indicators generally used in the monitoring and evaluation of contraceptive programmes.

### Data analysis

While all 208 indicators have utility in monitoring and evaluating contraceptive programming generally, the initial analysis process highlighted that many reveal no significant information with respect to human rights sensitivity. Consequently, 123 of the indicators were excluded, while 85 of the indicators were prioritized for in-depth review by the research team using the analytical approach reflected in S1 Table. To highlight programming gaps and strengths, these indicators were then organized into categories previously identified by WHO and UNFPA as relevant to the provision of primary health care and contraceptive information and services [11]:

1. Ensuring access for all
2. *Commodities*, *logistics & procurement*
3. *Organization of health facilities*: *outreach; integration*
4. Quality of care
5. Comprehensive sexuality education
6. Humanitarian context
7. Participation by potential and actual users of services
8. Accountability to those using services

Following this categorization, frequency analyses were conducted to identify key trends and gaps (full tables available upon request). These analyses determined:

1. the number of indicators within and across categories that explicitly or implicitly addressed human rights principles or standards;
2. the number of human rights addressed by the indicators within and across categories;
3. the number of indicators within and across categories that included a focus on specific populations and/or inequalities;
4. the number of indicators within and across categories that reflected user perspectives; and,
5. the number of indicators within and across categories that lent themselves to disaggregation and investigation for potential accordance with non-discrimination law.

## Findings/Results

This section presents illustrative findings only to give a sense of the sort of information this analysis can elicit; it does not constitute a systematic presentation of the findings. The relevance of this work to ensuring the human rights sensitivity of indicators used in public health more generally is explored in the Discussion section below.

The results of the above-described analyses were presented to a group of key stakeholders who ultimately prioritized 42 of the 85 indicators grouped according to the 8 categories described above. As the human rights sensitivity of the 85 indicators had already been established, prioritization of the 42 was based on a range of factors, including utility to decision-makers in monitoring and evaluating key aspects of contraceptive programming, data availability in terms of the frequency and feasibility that these data are collected, and complementarity with respect to indicators that could complement one another to provide the most holistic or complete assessment possible. It was recognized that no single existing indicator could adequately provide meaningful information on all health and human rights issues that are relevant to all aspects of contraceptive programmes, and that a range of indicators needs to be in place to truly capture the extent to which contraceptive programmes respect, protect and fulfill relevant rights. Of the final 42 indicators, 13 were quantitative, 7 were qualitative and 22 were policy-level [12].

Tables produced for each of the eight categories relevant to the delivery of contraceptive programmes were color-coded to aid in visual representation to highlight where human rights principles were addressed and where gaps existed. While some categories had a healthy mix of indicators, both with respect to type of indicator and to human rights concerns captured, others were extremely deficient. S2 Table illustrates this for select indicators under Category 2: ‘Commodities, Logistics & Procurement’.

Findings from this category illustrate a number of relevant issues. While some indicators address availability and accessibility, none address informed decision-making, privacy and confidentiality, or participation. Furthermore, only one indicator contains an explicit linkage to a human right (shaded in dark grey) while the rest only implicitly link to human rights (shaded in light grey). In addition, none of the indicators reflect user perspectives or lend themselves to disaggregation and investigation for potential accordance with non-discrimination law, with only one indicator that includes a focus on a specific population and/or inequalities (shaded in black).

**Fig 1:**
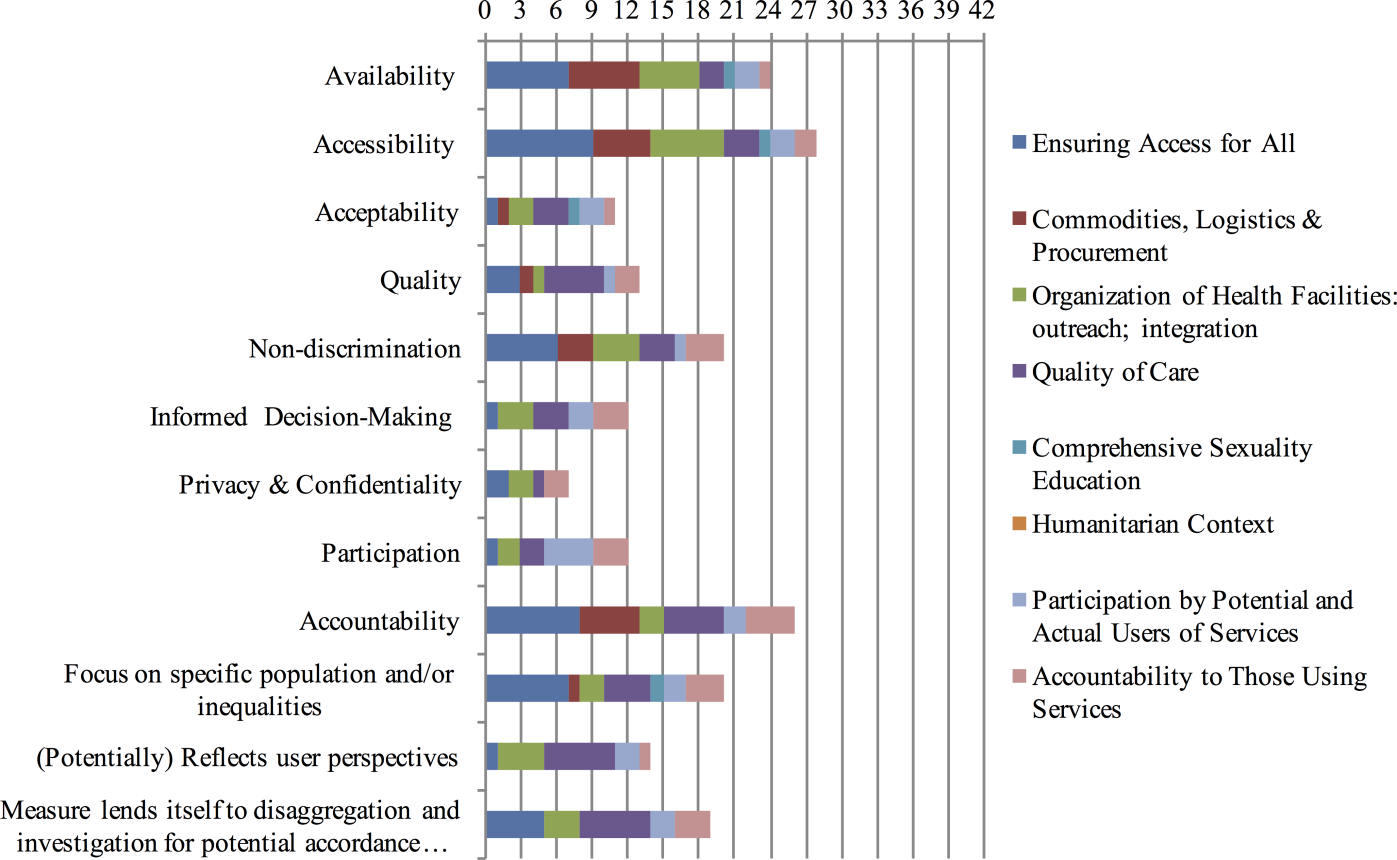
Number of indicators across all categories that address each human rights principle, standard and key criteria.

Overall, across categories, results of the frequency analysis yield the following information presented in Fig 1:

1. It is far more likely for indicators to implicitly rather than explicitly link to human rights;
2. The human rights principles most often addressed by the prioritized indicators are accessibility (n = 28), accountability (n = 26), availability (n = 24) and non-discrimination (n = 20);
3. Those least often addressed are informed decision-making (n = 12), participation (n = 12), acceptability (n = 11), and privacy and confidentiality (n = 7);
4. Roughly half of the prioritized indicators include a focus on a specific population and/or inequalities;
5. A third of these indicators reflect user perspectives; and,
6. Approximately half lend themselves to disaggregation and investigation for potential accordance with non-discrimination law.

Taken as a whole, findings from this analysis illustrate that while many indicators generally used to monitor contraception programmes have some degree of sensitivity to human rights, the breadth and depth one would expect are lacking (full results, including the final list of 42 prioritized indicators, are forthcoming in a WHO report).

## Discussion

Human rights are increasingly being brought into public health programming, but challenges remain in the dearth of public health indicators that sufficiently take human rights into account, the quality of data collected through routine monitoring as well as how data monitoring systems are configured more generally. The methodology presented here provides a series of steps to support the collection, analysis and ultimate prioritization of existing indicators used in public health programming based on their sensitivity to human rights principles and standards. The domains for analysis are designed to allow those who use it to systematically take stock of what indicators currently in use in a particular setting on a particular health topic address human rights concerns and to allow for identification of key gaps.

As was recognized in WHO work in relation to contraception, “Wherever possible, the disaggregation of information on the basis of sex, age, urban/rural residence, ethnicity, level of education, wealth quintile and geographic region is essential for ensuring non-discrimination and equity, and as a basis for affording due protection to vulnerable and marginalized groups.” Other considerations include the timeliness, scope and number of indicators used, and critically, the extent to which they incorporate human rights, either explicitly or implicitly. Despite the preference of large-scale initiatives for quantitative indicators, a purposeful combination of quantitative, qualitative, and policy indicators is critical to highlight rights concerns, drawing attention to a range of human rights issues that might not otherwise be captured. This includes any gaps between laws and policies, the ways in which relevant services are delivered, and how they are understood and used by their potential beneficiaries. Thus, any indicator set must to the extent possible attempt a solid mix.

A key issue to be addressed in any such exercise is how human rights are understood even if ostensibly included in programming and monitoring. Is the use of human rights in these efforts based on *explicit* attention to internationally recognized rights in the legal sense, or are rights (or a specific right) more *implicitly* recognized? The difference between what is explicit or implicit has implications for how implementers and evaluators understand each other, and for legal accountability, but it also has direct implications for monitoring. The concept of participation forms an illustrative example. *Explicit* attention to the right to participation means that in monitoring, it is possible to determine if the programme has steps in place to ensure the participation of relevant communities, has budgeted for their participation, holds participation as a desirable programme outcome, and if there is any legal and policy support to ensure participation of relevant communities in such activities. This cannot be done as clearly if the commitment to participation is more *implicitly* part of the programme’s ethos: capturing how the right to participation is implemented becomes a challenge with implications for choosing appropriate indicators.

User perspectives are also central because they bring to light people’s lived experiences around their use of health programmes and services, as well as people’s needs, preferences, and the acceptability of services offered. Inequalities within and across populations are of course important to take into account in determining whether health services and programmes are working for those most in need. Focusing on specific populations and potential inequalities in their access and use of services is also a corollary to the ability to disaggregate data by various factors that can help determine the existence and/or extent of discrimination, and can draw attention to the promotion or denial of human rights for specific populations in how relevant services are being delivered.

While the practical relevance and the feasibility of data disaggregation need to be appropriately addressed in each case with particular attention to statistical validity, disaggregation of data can help with the design, adaptation, implementation, and further monitoring of initiatives by pointing out where inequalities exist, and can contribute to the detection of related human rights concerns, including direct or indirect discrimination. While non-discrimination is a recognized human right most public health data are not disaggregated according to most recognized grounds of discrimination. While disaggregation should of course not happen at the expense of the statistical significance of the data collected, attention to the match between categories afforded protection under national non-discrimination law and disaggregated public health data has the potential to improve services, while supporting redress and accountability.

To enhance the value of an exercise such as this one, the disciplinary mix of the team doing this work is of critical importance. Engaged discussion combining knowledge of the specificities of each indicator with solid grounding in and knowledge of human rights is key. Once indicators are identified and relevant data collected and analyzed, this information should be presented to leadership as relevant to improve: training and capacity building of health workers and others involved in service provision; data monitoring; programming; and, development or modification of laws, policies, and protocols or guidelines.

**Fig 2:**
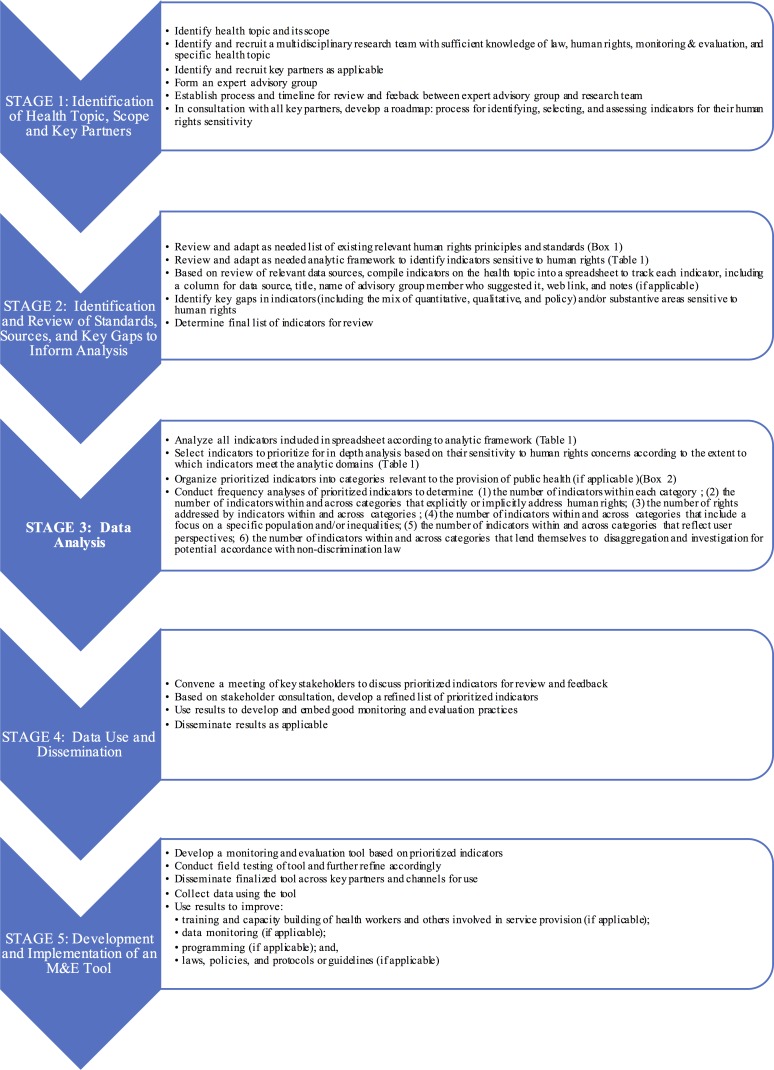
Steps to identify and Aasess human rights in public health indicators.

A high-level overview of the steps involved in such an exercise is presented below in Fig 2.

## Conclusion

While countries have increasingly made commitments to integrating human rights into their health programming, strong gaps remain in measuring how attention to human rights is incorporated into the access, use and delivery of public health programmes and services. It is hoped that this methodology can provide an approach for identifying the contribution of indicators generally used in public health programming, opening the way for follow-up actions sensitive to human rights concerns in any area of public health. Once relevant indicators have been identified and data collected, this information can help to identify gaps that exist where indicators remain to be developed, enhance accountability and be useful to the organizations involved in the planning, implementation, and/or evaluation of public health interventions, adding value to subnational and national reporting systems, as well as global initiatives. To increase the capacity for research, programming, measurement and accountability moving forward will require a re-configuration of health information management systems, related investments, and political will.

## Supporting information

S1 Table Information for inclusion and domains for indicator analysis. (TIFF)

S2 Table Analysis matrix showing links between select prioritized indicators in ‘Commodities, Logistics & Procurement’ and the health and human rights principles and standards key criteria. (TIFF)

S1 Appendix Relevant human rights principles and standards. (DOCX)

## References

[1] Bustreo F, Hunt P, Gruskin S, Eide A, McGoey L, Rao S, et al Women’s and children’s health: Evidence of impact of human rights. Geneva, Switzerland: World Health Organization, 2013.

[2] Sustainable Development Goals [Internet]. United Nations: The Sustainable Development Goals: 17 Goals to Transform our World; c2015. [cited 2016 Dec 13]. Available from: http://www.un.org/sustainabledevelopment/sustainable-development-goals/

[3] UN. Transforming our World: the 2030 agenda for sustainable development: A/RES/70/1. New York, NY: United Nations, 2015.

[4] Gruskin S, Ferguson L. Using indicators to determine the contribution of human rights to public health efforts. Bulletin of the World Health Organization. 2009; 87(9): 714–719. doi: 10.2471/BLT.08.058321 19784452 10.2471/BLT.08.058321 PMC2739915

[5] WHO. Leading the realization of human rights to health and through health. Report of the high-level working group on the health and human rights of women, children and adolescents. Geneva, Switzerland: World Health Organization; 2017.

[6] Report on 4th Meeting of Inter-Agency and Expert group on the Sustainable Development Goal Indicators. Geneva, Switzerland: United Nations Department of Economic and Social Affairs; 2016.

[7] Gruskin S, Waller E, Safreed-Harmon K, Ezer T, Cohen J, Gathumbi A et al Integrating human rights in program evaluation: Lessons from law and health programs in Kenya. Evaluation and social justice in complex sociopolitical contexts. 2015 doi: 10.1002/ev.20120

[8] Indicator and monitoring framework for the global strategy for women’s children’s and adolescents’ health. Geneva, Switzerland: World Health Organization; 2015.

[9] Gruskin S, Bogecho D, Ferguson L. ‘Rights-based approaches’ to health policies and programs: Articulations, ambiguities, and assessment. Journal of Public Health Policy. 2010 7;31(2):129–45. doi: 10.1057/jphp.2010.7 20535096 10.1057/jphp.2010.7

[10] WHO. Ensuring human rights within contraceptive service delivery: A human rights analysis of existing quantitative indicators. Geneva, Switzerland: World Health Organization; 2014.

[11] WHO. Ensuring human rights within contraceptive service delivery: Implementation guide. Geneva, Switzerland: World Health Organization; 2015.

[12] WHO. Monitoring human rights in contraceptive services and programmes. Geneva, Switzerland: World Health Organization; 2017.

