# Supplementary figures and images for: A novel methodology for strengthening human rights based monitoring in public health: Family planning indicators as an illustrative example
Source: PLoS One. 2017 Dec 8;12(12):e0186330. doi: 10.1371/journal.pone.0186330 (PMC5722344; doi:10.1371/journal.pone.0186330)

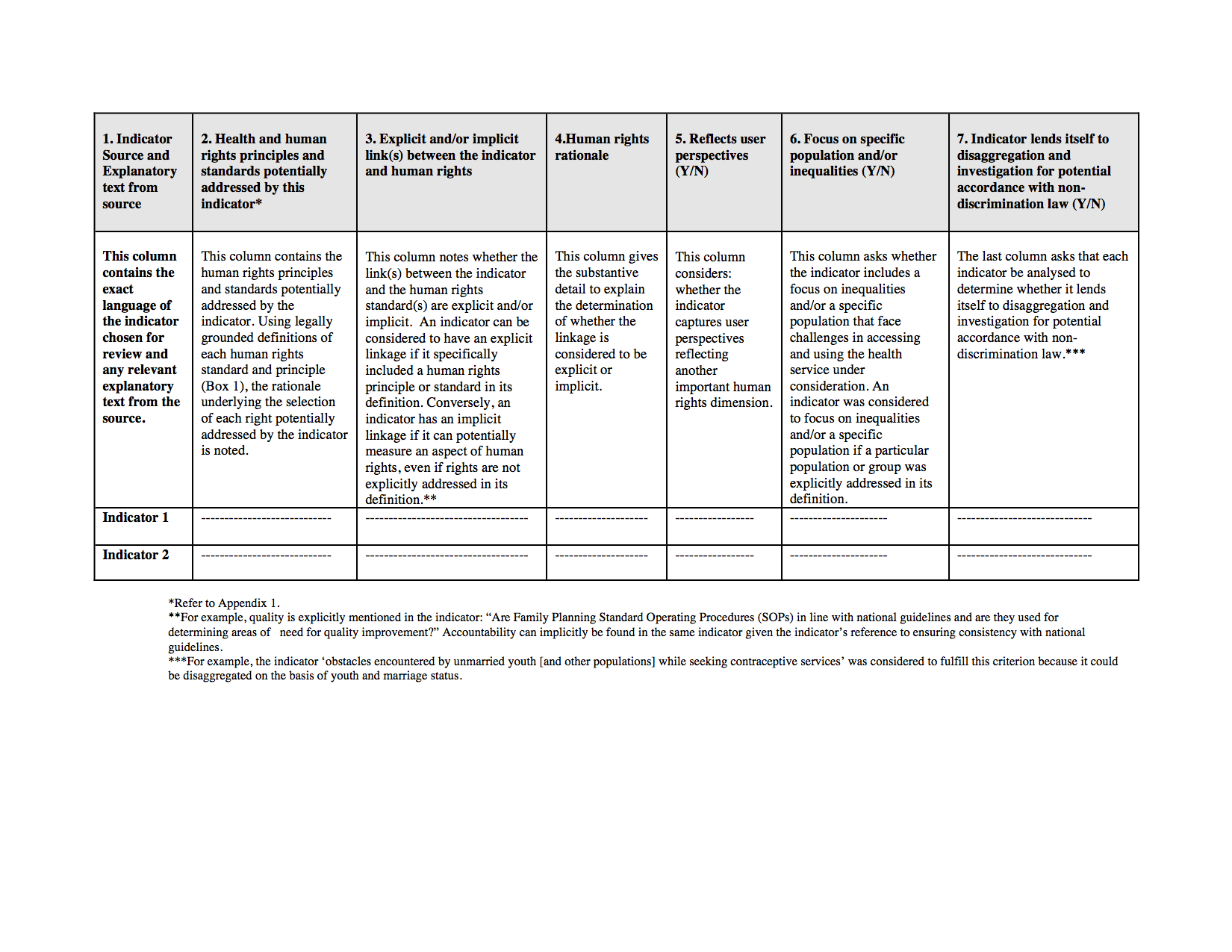

Supplement: S1 Table — (TIFF) [file pone.0186330.s001.tiff]

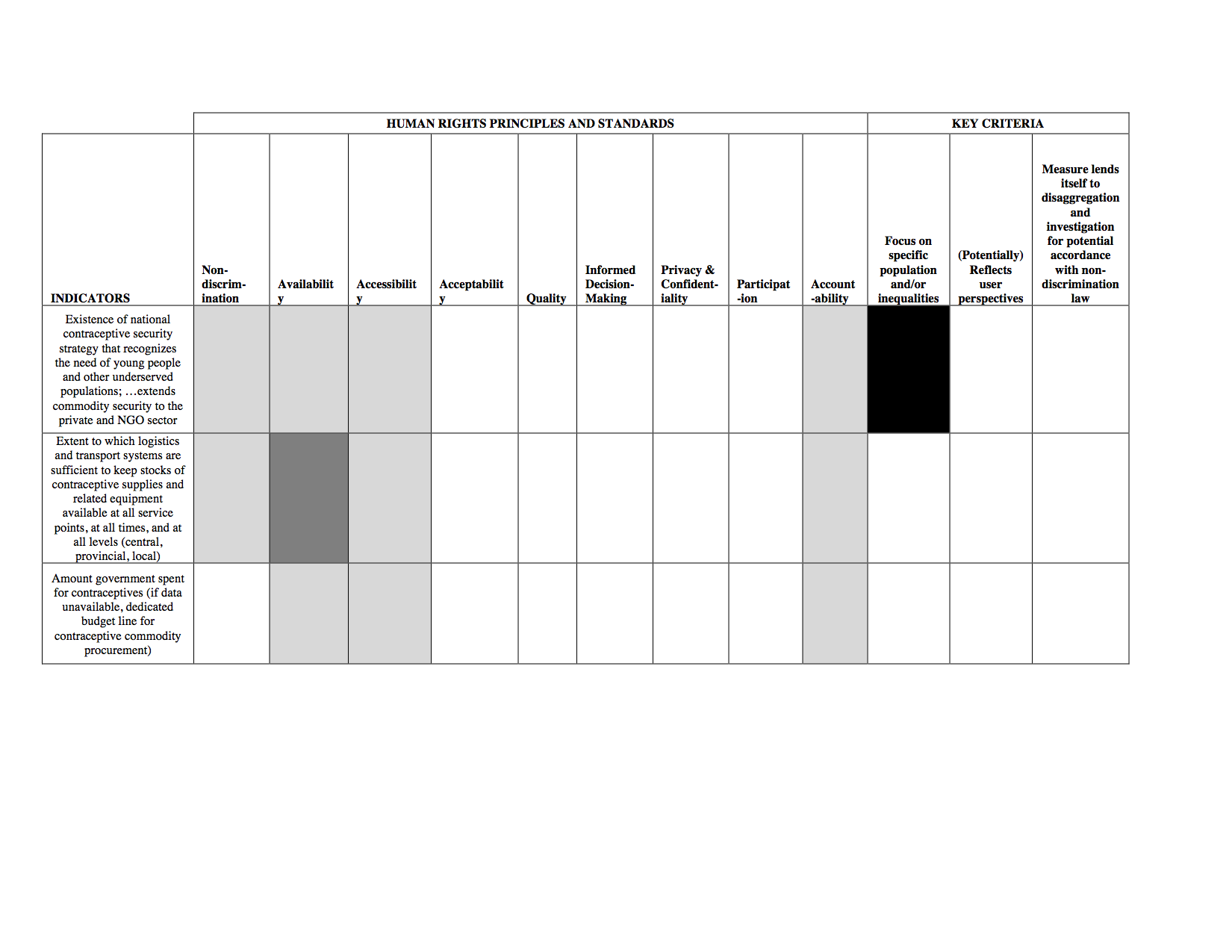

Supplement: S2 Table — (TIFF) [file pone.0186330.s002.tiff]
