# Supplementary material for: A novel methodology for strengthening human rights based monitoring in public health: Family planning indicators as an illustrative example
Source: PLoS One. 2017 Dec 8;12(12):e0186330. doi: 10.1371/journal.pone.0186330 (PMC5722344; doi:10.1371/journal.pone.0186330)
Supplement: S1 Appendix — (DOCX) [file pone.0186330.s003.docx]

**S1 Appendix: Relevant human rights principles and standards [11]**

| 1. **Non-discrimination**   The human rights principle of non-discrimination obliges States to guarantee that human rights are exercised without discrimination of any kind based on race, colour, sex, language, religion, political or other opinion, national or social origin, property, birth or other status such as disability, age, marital and family status, sexual orientation and gender identity, health status, place of residence, economic and social situation [12]. This obligation in connection with health programmes means countries are to ensure the availability, accessibility, acceptability and quality of services and information **without discrimination.** |
| --- |
| 1. **Right to Health**   **a) Availability**  Functioning health and health-care facilities, goods and services, as well as programmes, have to be **available** in sufficient quantity within the State [13]. The characteristics of the facilities, goods and services will vary depending on numerous factors, including the State’s developmental level. Countries must, however, address the underlying determinants of health, such as provision of safe and potable drinking water, adequate sanitation facilities, health-related education, hospitals, clinics and other health-related buildings, and ensure that trained medical and professional personnel are receiving domestically competitive salaries. As part of this core obligation, countries should ensure that the commodities listed in national formularies are based on the WHO Model List of Essential Medicines, which guides the procurement and supply of medicines in the public sector [13].  **b) Accessibility**  Under international human rights law, countries are required to ensure that health-care facilities, commodities and services are **accessible** to everyone. This includes physical and economic accessibility, as well as access to information [13]. Human rights bodies have called on countries to eliminate the barriers people face in accessing health services, such as high fees for services, the requirement for authorization by spouse, parent/guardian or hospital authorities, distance from health-care facilities, and the absence of convenient and affordable public transport [14].  **c) Acceptability**  All provision of health-care facilities, commodities and services must be **acceptable** to those who are their intended beneficiaries. They must be provided in a manner respectful of medical ethics and of the culture of individuals, minorities, peoples and communities; sensitive to gender and to life cycle requirements; must be designed to respect confidentiality, and improve the health status of those concerned [13]. Countries should place a gender perspective at the centre of all policies, programmes and services including planning, implementation and monitoring of such policies, programmes and services.  **d) Quality**  Fulfilment of human rights requires that health-care facilities, commodities and services be of good **quality**, and scientifically and medically appropriate. This requires, among other things, skilled medical personnel, scientifically approved and unexpired drugs and hospital equipment, safe and potable water, and adequate sanitation [13]. |
| 1. Informed decision-**making**   Respect for individual dignity and for the physical and mental integrity of each and every person using a health facility means also providing each person the opportunity to make health choices autonomously [14,15]. The principle of autonomy, expressed through **free, prior, full and informed decision-making**, is a central theme in medical ethics, and is embodied in human rights law [16]. People should be able to exercise their choice from across a range of options but also be free to refuse any and all options. In order to make an informed decision, comprehensive information, counseling and support should be made accessible for all people without discrimination, including young people, persons living with disabilities, indigenous peoples, ethnic minorities, people living with HIV, and transgender and intersex people [17]. |
| 1. **Privacy and confidentiality**   The right to privacy means that as and when an individual accessing health information and services, they should not be subject to interference with their privacy, and they should enjoy legal protection in this respect [18]. The health of an individual involves many sensitive issues that are not widely discussed within families or communities, and health workers are often entrusted with very personal information by their patients. Confidentiality, which implies the duty of providers to not disclose or to keep private the medical information they receive from patients and to protect an individual’s privacy, has an important role to play in people’s trust in a health system or service. |
| 1. **Participation**   Under international human rights law, countries have an obligation to ensure active, informed **participation** of individuals in decision-making that affects them, including on matters related to their health [13]. Participation of affected populations in all stages of decision-making, implementation and monitoring of policies, programmes and services is a precondition for sustainable development and the highest attainable standard of health [19,20]. Laws, policies and programmes better reflect the needs and perspectives of affected populations when members of these populations take part in their development, thus helping to secure improvements in health outcomes and the quality of health care [21,22]. Under international human rights law, countries have an obligation to ensure active, informed participation of individuals in decision-making that affects them, without discrimination, including on matters related to their health [13]. They also have the obligation to ensure the meaningful participation of adolescents in all policies and programmes affecting their health [23]. |
| 1. **Accountability**   Countries are accountable for bringing their legal, policy and programmatic frameworks and practices in line with international human rights standards [24]. Further, effective accountability mechanisms are key to ensuring that the agency and choices of individuals are respected, protected and fulfilled, including when seeking and receiving health care. Effective accountability requires individuals, families and groups, including those from marginalized populations, are made aware of their rights, including with regard to their health, and are empowered to claim their rights [25]. |

**Appendix References**

1. United Nations Committee on Economic, Social and Cultural Rights. General comment No. 20 (2009): Non-discrimination in economic, social and cultural rights (article 2, paragraph 2, of the International Covenant on Economic, Social and Cultural Rights)*.* Geneva, Switzerland: United Nations Economic and Social Council; 2009.
2. United Nations Economic and Social Council. General Comment No. 14: The Right to the Highest Attainable Standard of Health (Art. 12 of the Covenant). Geneva: UN Committee on Economic, Social and Cultural Rights (CESCR); 2000.

UN General Assembly. General recommendation No. 24 (20th session): Article 12 of the Convention on the Elimination of All Forms of Discrimination against Women (CEDAW) — women and health. Report of the Committee on the Elimination of Discrimination against Women, Fifty-fourth session of the General Assembly, Supplement No. 38 (Chapter I). New York (NY): United Nations; 1999 (A/54/38/ Rev.1).

CEDAW. United Nations Convention on the Elimination of All Forms of Discrimination against Women (CEDAW). New York (NY): United Nations General Assembly; 1979 (A/47/38, 1249 UNTS 14).

1. Faden RR, Beauchamp TL. A History and theory of informed consent. New York: Oxford University Press; 1986.
2. Sexual and reproductive health: Core competencies in primary care. Geneva, Switzerland: World Health Organization; 2011.
3. Office of the United Nations High Commissioner for Human Rights. International Covenant on Economic, Social and Cultural Rights; 1996. Available from: http://www.ohchr.org/EN/ProfessionalInterest/Pages/CESCR.aspx via the Internet. Accessed 20 December 2016.
4. UNDP. Investing in development: A Practical plan to achieve the millennium development goals. Report to the UN Secretary-General. New York (NY): UN Development Programme; 2005.

Bradley SEK, Schwandt HM, Khan S. Levels, trends, and reasons for contraceptive discontinuation. DHS Analytical Studies No. 20. Calverton (MD): ICF Macro; 2009.

Ferguson L, Halliday E. Participation and human rights: impact on women and children’s health. What does the literature tell us? In: Bustreo F, Hunt P, Gruskin S, Eide A, McGoey L, Rao S, et al. Women’s and children’s health: Evidence of impact of human rights. Geneva, Switzerland: World Health Organization, 2013.

1. Potts H. Participation and the right to the highest attainable standard of health. Colchester: University of Essex Human Rights Centre; 2008.
2. United Nations Committee on the Rights of the Child. General Comment No. 15 (2003): The right of the child to the enjoyment of the highest attainable standard of health (article 24 of the Convention on the Rights of the Child). New York (NY): United Nations; 2003. (CRC/GC/2003/4).
3. Commission on information and accountability for women’s and children’s health. Translating recommendations into action. First progress report on implementation of recommendations: November 2011–June 2012. Geneva, Switzerland: World Health Organization; 2012.
4. Technical guidance on the application of a human rights-based approach to the implementation of policies and programmes to reduce preventable maternal morbidity and mortality. Report of the Office of the United Nations High Commissioner for Human Rights (A/HRC/21/22). Human Rights Council (20th session). Geneva, Switzerland: United Nations General Assembly; 2012.
